# Workplace Violence as an Occupational Hazard in Psychiatric Nursing: Burnout, Quality of Life, and Turnover Intentions in Saudi Arabia

**DOI:** 10.3390/healthcare14142190

**Published:** 2026-07-20

**Authors:** Majed Mowanes Alruwaili

**Affiliations:** Department of Nursing Administration and Education, College of Nursing, Jouf University, Sakaka 72388, Saudi Arabia; majed@ju.edu.sa

**Keywords:** occupational health, worker safety, workplace violence, psychiatric nursing, workplace well-being, burnout, quality of life, turnover intentions, psychosocial risk

## Abstract

**Background/Objectives**: Workplace violence (WPV) is a major occupational health hazard and psychosocial safety concern in psychiatric nursing, yet evidence integrating exposure, worker well-being, and workforce stability within a single theory-informed model remains limited in Saudi Arabia. This study examined associations between WPV and proximal occupational outcomes (burnout, job satisfaction, and absenteeism) and distal outcomes (quality of life [QoL] and turnover intentions) among psychiatric nurses and tested burnout as a statistical mediator of the WPV–turnover intentions association. **Methods**: A multicentre cross-sectional census survey was conducted across three psychiatric facilities in northern Saudi Arabia between June and August 2025 (N = 171). Participants reported WPV exposure over the preceding 12 months using Arabic versions of standardised instruments, including validated Arabic versions where available and a forward–backward-translated WVSQ. Linear, negative binomial, and logistic regression models examined outcome associations and factors associated with WPV exposure; the PROCESS macro (Model 4, 5000 bootstrap resamples) tested statistical mediation. **Results**: Most nurses reported at least one WPV incident in the preceding 12 months, most commonly verbal abuse. Younger age, male sex, shorter psychiatric nursing experience, lower perceived staffing adequacy, and inadequate security were associated with higher WPV exposure. WPV exposure was associated with lower QoL across all WHOQOL-BREF domains, higher burnout, lower job satisfaction, higher absenteeism, and stronger turnover intentions. Burnout showed a partial statistical mediation pattern in the WPV–turnover intentions association. Because the study was not specifically powered for the indirect effect, the mediation result should be interpreted with caution. **Conclusions**: In this cross-sectional study, WPV was a pervasive occupational exposure among Saudi psychiatric nurses and was associated with poorer QoL and higher turnover intentions, with burnout showing a partial statistical mediation pattern. Multilevel prevention, safer staffing, and structured post-incident psychological support are needed to support psychiatric nurses’ well-being and workforce retention.

## 1. Introduction

Workplace violence (WPV) is a persistent occupational hazard in healthcare and a major threat to staff safety, psychological well-being, and workforce sustainability [1,2]. In healthcare settings, WPV includes verbal abuse, threats, intimidation, and physical assault perpetrated by patients, family members, visitors, or coworkers [3]. Psychiatric nursing is consistently recognised as a high-risk area because nurses provide prolonged, close-contact care in contexts marked by acute distress, behavioural dysregulation, psychosis, and substance-related crises [4,5]. This clinical reality increases repeated exposure to non-physical aggression as well as the risk of physical incidents, with effects that extend beyond immediate harm [6]. Over time, recurring WPV can erode professional functioning, compromise therapeutic engagement, and weaken continuity of care in mental health services [7,8]. Accordingly, WPV should be conceptualised not only as an interpersonal safety incident but also as an occupational health exposure with far-reaching consequences for workers’ psychosocial well-being, functional quality of life, and retention.

Globally, WPV has been framed not only as an occupational safety problem but also as a health system challenge, given its effects on absenteeism, reduced job satisfaction, burnout, and increased attrition risk [9]. In psychiatric services, these consequences are especially concerning because workforce stability is essential for therapeutic continuity, de-escalation quality, and patient safety [10]. Thus, understanding WPV in psychiatric nursing requires an integrated approach that links exposure patterns to both nurse well-being and workforce outcomes [11,12].

In Saudi Arabia, psychiatric nurses practice within a sociocultural and institutional context that may amplify both exposure risk and impact. Staffing constraints, variability in security measures, and differences in reporting and response practices may reduce protection and recovery capacity after violent events [13,14]. In parallel, stigma surrounding mental illness and heightened family stress during psychiatric crises may intensify conflict at the point of care [15]. These contextual features shape the occurrence, interpretation, reporting, and consequences of WPV [16]. Several country-specific features may distinguish the Saudi psychiatric workforce from those described in many Western systems and warrant dedicated study. First, the workforce is highly multinational and linguistically diverse, so that language and cultural distance between nurses, patients, and families can complicate de-escalation and incident reporting [14]. Second, mental illness stigma and help-seeking reluctance remain comparatively pronounced in the region, which may delay presentation, heighten acuity at the point of contact, and discourage formal reporting of violence [17,18]. Third, rapid service expansion under Vision 2030 health-system reform has increased demand on a relatively young psychiatric nursing cohort, among whom early-career turnover is already a recognised concern [19]. A recent systematic review confirmed a high pooled prevalence of WPV against nurses in Saudi Arabia, yet most regional evidence has aggregated general-ward populations and seldom isolated psychiatric nurses or linked exposure to downstream well-being and retention outcomes [2]. Despite increasing regional attention to occupational safety, available evidence remains fragmented and often insufficient for psychiatric workforce planning [19].

A key limitation in the existing literature is that WPV is frequently treated as a prevalence problem without adequate integration of downstream outcomes [19]. Many studies report incident rates but do not simultaneously evaluate how exposure relates to nurse-centred outcomes (e.g., quality of life and burnout) and workforce outcomes (e.g., absenteeism and turnover intentions) within a single analytic framework [7,20,21]. This creates an evidence gap: decision-makers may know how often WPV occurs, yet remain uncertain about the strength of its association with retention risk and broader occupational well-being [22]. A second gap is contextual transferability: findings from non-psychiatric or non-regional systems may not generalise to Saudi psychiatric care, where institutional safeguards and sociocultural norms differ [23]. A third gap is translational: associations are often reported without a coherent mechanism-based model that identifies practical intervention points for prevention, support, and retention policy [24].

Against this background, the specific contribution of the present study is fourfold. First, it focuses on psychiatric nurses rather than a mixed hospital workforce. Second, it is set across three psychiatric facilities in northern Saudi Arabia, a region that remains under-represented in the regional workplace-violence evidence base. Third, it examines quality of life and turnover intentions together as dual distal outcomes, alongside burnout, job satisfaction, and absenteeism as proximal occupational outcomes. Fourth, it tests burnout as a statistical mediation pathway linking WPV exposure with turnover intentions within a theory-informed research model. To our knowledge, these elements have not previously been integrated into a single analytic model for the Saudi psychiatric nursing workforce, which represents the central contribution of this study.

Quality of life (QoL) is particularly relevant in this context because it captures a multidimensional burden beyond incident counts or single-symptom domains [25]. From the WHOQOL perspective, QoL encompasses physical, psychological, social, and environmental domains, each of which may be adversely affected by WPV [26]. Repeated exposure may be associated with poorer QoL across several plausible pathways: sustained sympathetic and hypothalamic–pituitary–adrenal activation and sleep disruption may compromise physical health; intrusive recollection, hypervigilance, and emotional exhaustion may erode psychological health; avoidance and withdrawal from colleagues and family may weaken social relationships; and diminished perceptions of safety and organisational support may lower environmental QoL. Examining QoL alongside turnover intentions provides a strategically important dual endpoint: one reflects functional well-being, and the other reflects the risk of workforce instability. This dual-outcome focus is directly aligned with service-level priorities in psychiatric care, where safe staffing and continuity of skilled nursing are central to the quality of care [27,28].

In light of these gaps and the multidimensional nature of the exposure, the aim of the present study was to examine workplace violence against psychiatric nurses in Saudi Arabia and to evaluate its associations with turnover intentions and quality of life, alongside burnout, job satisfaction, and absenteeism.

Accordingly, the study addressed the following research question: What are the prevalence and patterns of workplace violence experienced by psychiatric nurses in Saudi Arabia, and how is this exposure associated with quality of life, burnout, job satisfaction, absenteeism, and turnover intentions? The theoretical framework and specific hypotheses that guided this enquiry are developed in the following section, and the study objectives that operationalise them are set out thereafter.

### Theoretical Background and Hypothesis Development

To move beyond description, the study was theoretically anchored rather than purely empirical. It drew on the Transactional Model of Stress and Coping [29,30] and the Cognitive Appraisal Theory of Emotions [31,32]. These complementary frameworks were selected because they explain how exposure to a stressful event may be shaped by threat appraisal, perceived controllability, coping resources, and organisational conditions. In the context of psychiatric nursing, workplace violence may therefore be understood not only as an external safety incident, but also as an occupational stressor whose consequences may vary according to exposure characteristics, individual resources, and workplace conditions. The frameworks informed the development of the conceptual model, variable selection, hypothesis specification, and interpretation of the findings; they were not tested as formal structural models in this cross-sectional study.

Guided by these frameworks, four hypotheses were developed and are introduced in turn. Workplace violence is a recurrent occupational stressor; when it is appraised as threatening and poorly controllable, it may deplete coping resources and manifest as emotional exhaustion and as behavioural withdrawal from work [8,10]. WPV exposure was therefore expected to be associated with greater burnout and with more frequent stress-related absence. Accordingly:

**H1.**  *Higher WPV exposure is associated with higher burnout and greater absenteeism.*

Repeated exposure to aggression may also erode nurses’ appraisal of the work environment as safe, supportive, and equitable—an appraisal that is central to job satisfaction. Where violence is frequent and organisational protection is perceived as limited, satisfaction with the job is likely to decline [7,9]. Accordingly:

**H2.**  *Higher WPV exposure is associated with lower job satisfaction.*

Because WPV is a multidimensional stressor, its correlates are expected to extend beyond proximal occupational outcomes to distal endpoints that capture functional well-being and workforce stability. Sustained threat appraisal and diminished perceptions of organisational support may be reflected in poorer quality of life across the physical, psychological, social, and environmental domains, and in a stronger intention to leave the post [4,5,13,28]. Accordingly:

**H3.**  *Higher WPV exposure is associated with poorer quality of life and stronger turnover intentions.*

Finally, the two frameworks position burnout as a plausible intervening state linking a chronic stressor to withdrawal cognitions: recurrent violence may be appraised as an uncontrollable demand that fosters exhaustion and reduced professional efficacy, which in turn are associated with stronger consideration of leaving [10,19]. Because the data are cross-sectional, this pathway was specified as a statistical (non-causal) indirect association rather than a demonstrated mechanism. Accordingly:

**H4.**  *Burnout statistically mediates the association between WPV exposure and turnover intentions.*

Within this theory-informed analytic structure, WPV exposure was treated as the primary predictor; burnout, job satisfaction, and absenteeism as proximal occupational outcomes; and quality of life and turnover intentions as distal outcomes. Demographic, professional, organisational, and sociocultural characteristics were examined as predictors or contextual correlates of WPV exposure and, where applicable, were included as covariates in adjusted analyses.

To address this aim and to operationalise the hypotheses developed above, the study pursued four objectives:

- To estimate the 12-month prevalence and types of WPV exposure (verbal abuse, threats, intimidation, and physical assault) among psychiatric nurses;
- To assess associations between WPV exposure and proximal occupational outcomes (burnout, job satisfaction, and absenteeism) (H1, H2);
- To evaluate associations between WPV exposure and distal outcomes (quality of life and turnover intentions), including the burnout-mediated pathway (H3, H4);
- To identify demographic, professional, organisational, and sociocultural factors associated with WPV exposure in psychiatric settings.

## 2. Materials and Methods

### 2.1. Research Design

This study used a cross-sectional, descriptive correlational design [33] to examine workplace violence (WPV) among psychiatric nurses in northern Saudi Arabia and its associations with turnover intentions, quality of life, and related occupational outcomes. The two theoretical frameworks informed variable selection and the interpretation of associations, but were not estimated as latent structural models. The broader study implementation period extended from April to August 2025. Activities conducted before ethical approval were limited to preparatory procedures, including institutional coordination, site communication, research assistant training, and preparation of study materials. Participant recruitment, informed consent, questionnaire administration, and participant-level data collection were conducted between June and August 2025, after ethical approval had been granted. The WPV instrument asked participants to report exposure during the preceding 12 months, consistent with the standard recall period for workplace-violence surveys. Quality of life was assessed using the standard WHOQOL-BREF recall format. Measures and the analytic operationalisation of all key variables are described in Section 2.5.

### 2.2. Setting and Participants

The study was conducted across three psychiatric care facilities in northern Saudi Arabia. The final sample included nurses from a comprehensive mental health complex (n = 67), a specialised psychiatric hospital (n = 68), and a community-based mental health facility (n = 36). These facilities serve urban and semi-urban populations and provide inpatient, outpatient, emergency, and follow-up psychiatric services.

To maximise representativeness, a total enumeration (census) sampling strategy was employed [34]. The accessible population comprised all registered nurses actively engaged in direct patient care across the three facilities (N = 200). Inclusion criteria were: (1) registered nurse; (2) currently providing direct patient care in psychiatric services; and (3) at least 6 months of experience in the current role. Exclusion criteria were: (1) exclusively administrative duties; (2) extended leave during the data-collection period; and (3) temporary or agency employment not permanently assigned to the facility.

Recruitment and non-response. At the commencement of recruitment, 200 nurses met the eligibility criteria and were invited to participate. Of these, 171 provided informed consent and completed the study procedures, yielding a response rate of 85.5%. The 29 eligible nonparticipants comprised 18 nurses who declined participation and 11 who became unavailable after invitation because they commenced extended leave or could not be reached during the fieldwork period. To assess potential non-response bias, participants and eligible nonparticipants were compared using available staffing-roster data on age band, sex, and facility. No statistically significant differences were identified between the two groups for these characteristics (all *p* > 0.05), providing no evidence of differential non-response according to the available variables, although unmeasured differences cannot be excluded. An a priori power analysis conducted using G*Power (version 3.1.9.7 Düsseldorf, Germany), assuming a medium effect size (f^2^ = 0.15), 80% power, and a two-sided α of 0.05 for multiple linear regression, indicated a minimum required sample of 120 participants. The final sample of 171 exceeded this requirement and was considered adequate for the planned primary multivariable regression analyses. Because this calculation was not specifically designed to establish power for the indirect effect, the mediation findings were interpreted cautiously, particularly given the cross-sectional design.

The demographic and professional characteristics of the 171 participating nurses are summarised in Table 1. The sample was predominantly female (82.5%); the median age was 35 years (IQR 29–43), and the mean duration of psychiatric nursing experience was 8.5 years (SD = 5.2). Most participants held a bachelor’s degree (58.5%), and participants were distributed across the three facilities described above.

### 2.3. Data-Collection Procedure

Data collection followed a standardised three-phase protocol. During the preparatory phase, institutional coordination was completed at each site. Before fieldwork, research assistants received protocol-based training on participant approach, informed consent, questionnaire administration, confidentiality safeguards, and data handling. This training did not involve contact with eligible participants or the collection of participant-level data. The field phase commenced only after approval was granted by the Bioethics Institutional Review Board at Jouf University on 1 June 2025. Eligible nurses were approached during scheduled shifts and provided with written and verbal information about the study. After providing written informed consent, participants completed the paper-based questionnaire package in designated private areas within the facilities. Participation status and responses were not disclosed to supervisors or managers. During the data-management phase, completed questionnaires were returned in sealed envelopes and collected in secure boxes. Each participant was assigned a unique non-identifiable code. Data were double-entered and cross-verified, and quality-control procedures included range checks, logic-consistency screening, and outlier inspection before analysis.

### 2.4. Ethical Considerations and Anonymisation

The study was approved by the Bioethics Institutional Review Board at Jouf University (Approval No. 7522-2025; approval date 1 June 2025) and conducted in accordance with the Declaration of Helsinki. No participant recruitment, informed consent, questionnaire administration, or participant-level data collection occurred before IRB approval. Written informed consent was obtained from all participants prior to inclusion.

### 2.5. Measures and Operationalisation

Variable selection and operationalisation were guided by the conceptual framework: WPV exposure was specified as the primary predictor; burnout, job satisfaction, and absenteeism as proximal occupational outcomes; and quality of life and turnover intentions as distal outcomes. Contextual factors and demographic and professional characteristics were examined as predictors or contextual correlates of WPV exposure and, where applicable, were included as covariates in adjusted models. Internal-consistency reliability (Cronbach’s α) obtained in the present sample is reported with each instrument.

#### 2.5.1. Workplace Violence Exposure (Primary Predictor)

WPV was measured using the Workplace Violence Survey Questionnaire (WVSQ), developed within the ILO/ICN/WHO/PSI framework [35]. The 32-item instrument assesses four domains: physical assault, verbal abuse, threats, and intimidation (8 items per domain). Participants reported both the occurrence (yes/no) and the frequency (5-point Likert scale) of incidents during the preceding 12 months. Domain and total scores were computed according to instrument guidance, with higher scores indicating greater cumulative exposure burden. For descriptive analyses, WPV was dichotomised (any vs. no exposure in the past 12 months), whereas regression models treated the WPV total score as a continuous variable. An Arabic version was prepared for this study through the forward–backward translation and expert-panel procedure detailed in Section 2.6; cultural adaptation was limited to ensuring linguistic equivalence and adding context-appropriate examples, with no items added or removed. Internal consistency in the present sample was high (Cronbach’s α = 0.93 for the total scale; subscales 0.84–0.90).

#### 2.5.2. Quality of Life (Distal Outcome)

Multidimensional quality of life was assessed using the WHOQOL-BREF (26 items) [36]. The instrument covers four domains: physical health (7 items), psychological health (6 items), social relationships (3 items), and environment (8 items), plus two general items on overall QoL and satisfaction with health. Items are rated on 5-point scales and transformed to 0–100 domain scores following WHO scoring procedures; higher scores indicate better quality of life [37,38]. A previously validated Arabic version was used; in the present sample, internal consistency was acceptable to good (overall α = 0.89; domain α = 0.74–0.86). In the analyses, QoL was treated as a continuous distal outcome.

#### 2.5.3. Burnout (Proximal Outcome)

Occupational burnout was measured using the Copenhagen Burnout Inventory (CBI; 19 items), which assesses three domains: personal, work-related, and client-related burnout [39]. Items are rated on a 5-point response scale and transformed to a 0–100 metric per domain, with higher scores indicating greater burnout. In the present analyses, a global burnout score was calculated as the mean of the three domain scores, yielding a 0–100 score. This approach was chosen to provide a single interpretable burnout term for the multivariable and mediation models while preserving the conventional CBI metric. The three CBI domains were highly inter-correlated in this sample (r = 0.62–0.74) and showed high internal consistency (global score α = 0.94; domains 0.83–0.90). Domain scores were additionally examined as continuous proximal outcomes, and the substantive findings were unchanged.

#### 2.5.4. Job Satisfaction (Proximal Outcome)

Job satisfaction was assessed using the Job Satisfaction Survey (JSS), a 36-item instrument covering nine facets: pay, promotion, supervision, fringe benefits, contingent rewards, operating procedures, coworkers, nature of work, and communication [40,41,42]. Items are scored on a 6-point Likert scale (1 = strongly disagree to 6 = strongly agree). Total scores range from 36 to 216, with higher scores indicating greater job satisfaction. Conventional cut-offs (36–108 = dissatisfied, 109–144 = ambivalent, 145–216 = satisfied) were applied for descriptive summaries, whereas all inferential and multivariable analyses treated the JSS total score as a continuous proximal outcome. Internal consistency in the present sample was high (α = 0.91).

#### 2.5.5. Workforce Outcomes (Proximal and Distal)

Two workforce-related indicators were included. Absenteeism (proximal outcome) was measured as the self-reported number of workdays missed due to work-related stress or work-related injury during the previous 12 months and was analysed as a count outcome (see Section 2.7). Turnover intentions (distal outcome) were measured using the Turnover Intention Scale-6 (TIS-6), a 6-item instrument rated on a 5-point Likert scale (total score range 6–30); higher scores indicate stronger intention to leave the current job [43]. Conventional categories (low, moderate, high risk) were used descriptively, and multivariable models treated the TIS-6 total score as a continuous distal outcome. Internal consistency for the TIS-6 was good (α = 0.87).

#### 2.5.6. Contextual Factors and Covariates

Perceived contextual contributors to WPV were assessed using a structured questionnaire developed for this study, comprising 45 items across three domains: cultural factors (15 items), social factors (15 items), and systemic/organisational factors (15 items). Items are rated on a 5-point Likert scale (1 = strongly disagree to 5 = strongly agree), and domain scores were computed so that higher scores indicate a stronger perceived contribution to WPV risk. Items were generated from a structured review of the WPV-determinants literature and from the ILO/ICN/WHO/PSI framework, then mapped to the three a priori domains. Content validity was established by an expert panel of six reviewers (two psychiatric nursing academics, two senior psychiatric staff nurses, one occupational health physician, and one health services researcher), each of whom rated every item for relevance on a 4-point scale. Items with an item-level Content Validity Index (I-CVI) ≥ 0.78 were retained; three items below this threshold were revised and re-rated. The scale-level Content Validity Index (S-CVI/Ave) was 0.89. Internal consistency in the present sample was good (total α = 0.90; cultural α = 0.82, social α = 0.81, systemic/organisational α = 0.88).

Demographic and professional covariates included age, sex, marital status, years of nursing experience, years in psychiatric nursing, facility (a three-site indicator), primary work setting (e.g., inpatient vs. outpatient), and shift-related factors (e.g., rotation, night-shift involvement). These variables were included as adjustment factors in multivariable analyses.

### 2.6. Translation, Cultural Adaptation, and Pilot Testing

Validated Arabic versions of the standardised instruments were used where available (WHOQOL-BREF, CBI, JSS, and TIS-6). For the WVSQ and the study-specific contextual-factors questionnaire, which required contextual adaptation, a standard forward–backward translation procedure was applied: two independent bilingual translators produced forward Arabic translations, a third bilingual translator blind to the original performed the back-translation, and discrepancies were reconciled by the expert panel described in Section 2.5.6 to confirm semantic, idiomatic, and contextual equivalence. This adaptation was conducted as part of the present study rather than drawn from a prior validation. A pilot test (n = 15 psychiatric nurses) assessed comprehensibility, cultural relevance, and completion time; pilot participants were not included in the main sample. As reported with each instrument in Section 2.5, internal-consistency reliability (Cronbach’s α) was evaluated for all multi-item scales in the main study, and all coefficients exceeded 0.70, indicating acceptable-to-excellent internal consistency.

### 2.7. Statistical Analysis

Analyses were performed using IBM SPSS Statistics Version 26.0 (IBM, NY, USA), with the PROCESS macro (v4.2) for mediation. Statistical significance was set at *p* < 0.05 (two-tailed). Descriptive and bivariate analyses. Frequencies, percentages, means, standard deviations, medians, and interquartile ranges characterised participants, WPV exposure, and scale scores. Chi-square tests and independent-samples *t*-tests (Welch’s correction for unequal group sizes) examined group differences (any vs. no WPV exposure), and Pearson correlations examined linear relationships among continuous variables. A total of seven pre-specified group comparisons were conducted (the four WHOQOL-BREF domain scores and overall quality of life, global burnout, and total job satisfaction; see Section 3.3. Given this limited number of pre-specified comparisons and the large observed effect sizes, no formal correction for multiple comparisons was applied; nevertheless, all reported group differences remained statistically significant at a Bonferroni-adjusted threshold (α = 0.05/7 = 0.007). Multivariable modelling. In line with the conceptual framework, multiple linear regression examined predictors of turnover intentions (WPV exposure, burnout, and job satisfaction), and logistic regression identified demographic, professional, and contextual factors associated with any WPV in the previous 12 months. Because absenteeism was an over-dispersed count (its variance exceeded its mean, and a likelihood-ratio test favoured the negative binomial over a Poisson specification), it was modelled primarily with negative binomial regression (reported as incidence-rate ratios); a linear model is additionally presented for comparability with the turnover model, and the two approaches yielded the same pattern of significant associations.

Covariate adjustment and confidence intervals. To assess the robustness of the theory-driven models, sensitivity analyses were conducted with adjustment for age, sex, years of psychiatric experience, and facility. WPV and burnout remained statistically significant, with materially unchanged coefficients; the adjusted estimates are reported in Section 3.5. Unstandardised coefficients are reported with 95% confidence intervals throughout (see Section 3.5 and Section 3.8). Common method bias. Because predictors and outcomes were self-reported in a single administration, Harman’s single-factor test was conducted; the first unrotated factor accounted for 28.4% of the variance (below the 50% threshold), indicating no evidence of a dominant single-factor structure; however, common method variance cannot be fully excluded.

Regression assumptions (linearity, normality of residuals, homoscedasticity, and multicollinearity) were assessed; variance inflation factors were below 2.0, indicating no problematic multicollinearity. Missing data were minimal (<5%) and handled using complete-case analysis for primary models. Effect sizes were reported as Cohen’s d for *t*-tests and standardised β values for regression models; unstandardised regression coefficients were presented with 95% confidence intervals. Mediation analysis. A theory-driven mediation model (PROCESS Model 4) tested whether burnout statistically mediated the WPV–turnover intentions association; indirect effects used 5000 bootstrap resamples and were considered significant when the 95% bias-corrected confidence interval excluded zero. Given the cross-sectional design, all mediation results are interpreted as statistical (non-causal) indirect associations, and temporal ordering among WPV, burnout, and turnover intentions cannot be established.

## 3. Results

The Results are organised by study objective and hypothesis. Section 3.1 addresses Objective 1 (the prevalence and types of WPV); Section 3.2 addresses Objective 4 (predictors of exposure); Section 3.3, Section 3.4 and Section 3.5 address Objectives 2–3 and Hypotheses H1–H3 (associations with proximal and distal outcomes); Section 3.6 reports the common-method-bias check; Section 3.7 reports the contextual factors associated with WPV; and Section 3.8 addresses Hypothesis H4 (the burnout-mediated pathway). Participant characteristics are reported in the Methods (Section 2.2, Table 1). Supporting analyses are presented sequentially in Supplementary Tables S1–S5.

### 3.1. Prevalence and Types of Workplace Violence

Overall, 140 nurses (81.9%; 95% CI 75.4–87.0) reported exposure to at least one form of WPV during the previous 12 months (Table 2). Verbal abuse was the most frequently reported form of WPV (76.0%; 95% CI 68.9–82.1), followed by threats (52.6%), intimidation (40.9%), and physical assault (29.2%). Non-physical forms of violence therefore predominated, although nearly one in three nurses reported at least one physical assault. Because categories were not mutually exclusive, participants could report more than one type of WPV. Consistent with the WVSQ recall format, exposure was assessed by type and frequency over the preceding 12 months; however, a detailed event-level breakdown by perpetrator and location was beyond the scope of the present instrument and is identified as a direction for future research.

### 3.2. Demographic and Professional Predictors of WPV Exposure (Objective 4)

As a planned objective, demographic, professional, and contextual factors associated with any WPV exposure were examined. Bivariate analyses indicated that younger nurses, male nurses, and nurses with fewer years of psychiatric nursing experience were more likely to report WPV in the previous 12 months. Detailed cross-tabulations of WPV types by age group, sex, and psychiatric nursing experience are provided in Supplementary Table S1. In multivariable logistic regression (Supplementary Table S2), younger age (20–30 vs. 51–60 years; OR = 2.50), male sex (OR = 2.00), and fewer than 5 years of psychiatric nursing experience (OR = 3.00) were associated with higher odds of reporting any WPV exposure. Contextual factors associated with WPV exposure (perceived staffing adequacy, security measures, and stigma toward mental illness) are reported separately in Section 3.7.

### 3.3. Differences in Quality of Life, Burnout, and Job Satisfaction by WPV Exposure

To address Objective 2 and Hypotheses H1–H3, quality of life, burnout, and job satisfaction were compared between nurses who reported WPV exposure in the previous 12 months and those who reported no WPV exposure. WPV-exposed nurses had significantly lower quality-of-life scores across all WHOQOL-BREF domains and lower job satisfaction than non-exposed nurses. They also had significantly higher global burnout scores. All between-group differences were statistically significant, with large effect sizes (Cohen’s d = 1.15–2.43), indicating both statistical and practical relevance (Table 3).

### 3.4. Correlations Between WPV, Well-Being, and Workforce Outcomes

Pearson correlations showed statistically significant associations among WPV exposure, well-being indicators, and workforce outcomes (Table 4). WPV exposure was positively correlated with burnout (r = 0.72), absenteeism (r = 0.60), and turnover intentions (r = 0.67), and negatively correlated with overall QoL (r = −0.65) and job satisfaction (r = −0.68). Burnout was positively correlated with absenteeism (r = 0.65) and turnover intentions (r = 0.70), and negatively correlated with overall QoL (r = −0.70) and job satisfaction (r = −0.62). Overall QoL and job satisfaction were inversely associated with absenteeism and turnover intentions. All correlations were statistically significant at *p* < 0.01.

### 3.5. Multivariable Models Predicting Absenteeism and Turnover Intentions

To address Objectives 2–3 and Hypotheses H1–H3, multivariable models examined associations with absenteeism and turnover intentions (Table 5). Because absenteeism was a count outcome, negative binomial regression was specified as the primary count model; the linear regression model is presented for comparability with the turnover-intentions model. In Model A, higher WPV exposure and higher global burnout were associated with more self-reported absence days. The negative binomial specification showed a consistent pattern of association for WPV exposure (incidence-rate ratio = 1.29, 95% CI 1.14–1.46). In Model B, higher WPV exposure and higher global burnout were associated with stronger turnover intentions, whereas higher job satisfaction was associated with lower turnover intentions. The turnover-intentions model explained 45% of the variance. Coefficients were materially unchanged after adjustment for age, sex, years of psychiatric nursing experience, and facility (Supplementary Table S5).

**Table 5:** Multivariable regression models predicting absenteeism and turnover intentions with 95% confidence intervals.

Model/Predictor	B	SE B	β	95% CI	t	*p*
Model A: Absenteeism						
WPV exposure	0.35	0.10	0.30	0.15–0.55	3.50	<0.001
Global burnout	0.75	0.24	0.28	0.27–1.23	3.12	0.002
Model fit	R^2^ = 0.40; Adj. R^2^ = 0.39; F(2, 168) = 56.00, *p* < 0.001					
Model B: Turnover intentions						
WPV exposure	0.40	0.09	0.35	0.22–0.58	4.44	<0.001
Global burnout	0.90	0.21	0.32	0.48–1.32	4.29	<0.001
Job satisfaction	−0.25	0.08	−0.27	−0.41 to −0.09	−3.13	0.002
Model fit	R^2^ = 0.45; Adj. R^2^ = 0.44; F(3, 167) = 45.55, *p* < 0.001					

Notes: Model A examined WPV exposure and global burnout as predictors of absenteeism. Because absenteeism was a count outcome, negative binomial regression was specified as the primary count model and produced a consistent pattern of association (WPV exposure incidence-rate ratio = 1.29, 95% CI 1.14–1.46); the linear model is shown for comparability. Model B examined WPV exposure, global burnout, and job satisfaction as predictors of turnover intentions. B = unstandardised coefficient; SE B = standard error; β = standardised coefficient; CI = confidence interval; WPV = workplace violence. Variance inflation factors were ≤2.0. Covariate-adjusted estimates are reported in Supplementary Table S5. The WPV coefficient in Model B (B = 0.40) is adjusted for both global burnout and job satisfaction and therefore differs from the unadjusted total effect of WPV on turnover intentions reported in the mediation model (Table 6, B = 0.70), which does not adjust for job satisfaction.

**Table 6:** Mediation analysis of burnout in the association between WPV exposure and turnover intentions.

Path	B	SE	95% CI	*p*
Total effect: WPV exposure → turnover intentions	0.70	0.09	0.52–0.88	<0.001
Direct effect: WPV exposure → turnover intentions, controlling for burnout	0.45	0.08	0.29–0.61	<0.001
Indirect effect: WPV exposure → burnout → turnover intentions	0.25	0.05	0.16–0.36	—

Note: Indirect effects were estimated using the PROCESS macro Model 4 with 5000 bootstrap resamples. The indirect effect was interpreted using the 95% bootstrap confidence interval; the confidence interval did not include zero, supporting a statistically significant indirect association. Mediation is interpreted as non-causal because of the cross-sectional design. The total effect of WPV exposure on turnover intentions (B = 0.70) is larger than the WPV coefficient in the multivariable turnover model (Table 5, Model B, B = 0.40) because the mediation model includes only burnout as the intervening variable, whereas Model B additionally adjusts for job satisfaction; the proportion of the association mediated by burnout (35.7%) should therefore be regarded as approximate.

In other words, holding the other predictors constant, nurses who experienced more workplace violence and greater burnout tended to miss more workdays and to report a stronger intention to leave their current post, whereas more satisfied nurses reported weaker turnover intentions. The count model indicated that higher WPV exposure corresponded to an approximately 29% higher rate of stress-related absence (IRR = 1.29), and burnout accounted for a meaningful share of the WPV–turnover association (Section 3.8). Because burnout and job satisfaction are potentially modifiable, these patterns suggest that lowering violence exposure while supporting staff well-being could be accompanied by reduced absence and stronger retention—an interpretation offered as a direction for intervention research rather than as evidence of cause, given the cross-sectional design.

### 3.6. Common Method Bias

Because all measures were self-reported in a single administration, Harman’s single-factor test was performed. The first unrotated factor accounted for 28.4% of the total variance, indicating no evidence of a dominant single-factor structure; however, common method variance cannot be fully excluded.

### 3.7. Contextual Factors Associated with WPV

Mean scores for perceived cultural, social, and systemic contributors to WPV are presented in Supplementary Table S4. Nurses reported high perceived contributions of staffing insufficiency, security limitations, and stigma related to mental illness. In the logistic regression model (Supplementary Table S2), lower perceived staffing adequacy, inadequate security, and higher perceived stigma toward mental illness were associated with higher odds of WPV exposure, whereas higher perceived staffing adequacy and adequate security were associated with lower odds of WPV exposure.

### 3.8. Burnout as a Statistical Mediator of the WPV–Turnover Intentions Association

Mediation analysis tested Hypothesis H4, namely whether burnout statistically mediated the association between WPV exposure and turnover intentions (Table 6). WPV exposure was positively associated with burnout, and burnout was positively associated with turnover intentions while controlling for WPV exposure. The total effect of WPV exposure on turnover intentions was significant (B = 0.70, 95% CI 0.52–0.88), and the indirect effect through burnout was statistically supported because the 95% bootstrap confidence interval excluded zero (B = 0.25, 95% bootstrap CI 0.16–0.36). The direct effect remained significant after accounting for burnout (B = 0.45, 95% CI 0.29–0.61). The indirect effect accounted for 35.7% of the total effect, indicating partial statistical mediation. Because of the cross-sectional design, this mediation finding is interpreted as a statistical indirect association rather than evidence of causal sequencing. The larger total effect in this model (B = 0.70) relative to the WPV coefficient in Table 5 (Model B, B = 0.40) reflects that Model B additionally adjusts for job satisfaction, whereas the mediation model includes only burnout as the intervening variable; the proportion of the association attributed to burnout (35.7%) should therefore be regarded as approximate.

## 4. Discussion

This theory-informed cross-sectional study examined workplace violence (WPV) against psychiatric nurses in Saudi Arabia and its associations with quality of life, burnout, job satisfaction, absenteeism, and turnover intentions. The findings indicate that WPV is not an occasional disturbance but a pervasive occupational exposure with measurable associations with both nurse well-being and workforce stability. The findings are interpreted below in relation to the four study hypotheses (H1–H4) and to prior Saudi, regional, and international evidence.

In this sample, 81.9% of psychiatric nurses reported at least one form of WPV in the preceding 12 months, with verbal abuse most frequent, followed by threats, intimidation, and physical assault. This prevalence is consistent with the pooled estimate reported for nurses in Saudi Arabia in a recent meta-analysis [2] and with psychiatric-nurse-specific findings from Saudi mental health hospitals [13]. It also falls within the wide international range reported for psychiatric nursing settings, while the predominance of verbal over physical aggression is consistent with broader regional evidence showing that verbal violence is more frequent than physical violence [44,45].

Repeated exposure at such levels risks a process of normalisation in which WPV is appraised as an expected, inevitable aspect of the role rather than as an unacceptable threat to safety. Within the Cognitive Appraisal Theory of Emotions [46], this can be interpreted as a distortion of primary appraisal: violent events are no longer consistently evaluated as critical stressors that warrant protective organisational responses [47]. When WPV is appraised as “part of the job,” incident reporting may decline, institutional urgency may be blunted, and opportunities for prevention and recovery may be missed. For psychiatric nursing management, recognising this normalisation process is essential to reframing WPV as a preventable occupational hazard rather than an inherent characteristic of psychiatric care [48,49,50]. In practical terms, reframing normalisation requires concrete managerial action rather than exhortation: for example, embedding brief structured debriefs after every reported incident, publishing unit-level incident dashboards so that violence is visible to staff and leaders, recognising and protecting time for reporting, and incorporating WPV indicators into ward performance reviews.

Younger nurses and those with fewer than 5 years of psychiatric experience were significantly more likely to report WPV. From the perspective of the Transactional Model of Stress and Coping, this pattern can be understood in terms of de-escalation self-efficacy and the availability of coping resources. Nurses with limited psychiatric experience may feel less confident in their ability to anticipate and de-escalate violent situations, appraise incidents as more threatening, and have fewer established formal or informal supports, increasing their vulnerability to both exposure and its psychological impact [51].

Male nurses showed higher odds of experiencing WPV than female nurses. Rather than attributing this solely to individual characteristics, the pattern is plausibly linked to the systemic allocation of risk: in many psychiatric units, male nurses are more frequently assigned to higher-acuity situations, including physical containment, management of acute agitation, and seclusion. This interpretation is consistent with external evidence: staff burnout and job dissatisfaction have been associated with violence exposure in psychiatric and healthcare settings [52,53]. Research among male nurses—including studies of psychiatric emergency and seclusion practice and of workplace bullying and work engagement—indicates that men are more often involved in high-acuity situations such as physical restraint and management of acute agitation [54,55,56]. Although the present data cannot directly observe assignment practices, the combination of elevated odds among male nurses and the nature of psychiatric work supports a system-level rather than purely individual interpretation. This system-level account is a post hoc interpretation that was not pre-specified and cannot be tested with the present cross-sectional data; it is therefore offered as a hypothesis for future research rather than as a confirmed explanation. Future qualitative work could clarify how such task allocation is negotiated in Saudi psychiatric settings.

Contextual factors further shaped WPV risk. Lower perceived staffing and inadequate security were associated with higher WPV occurrence, whereas better perceived staffing and security appeared protective, consistent with international guidance identifying staffing adequacy, environmental design, and security presence as central organisational levers for prevention [1,11]. Higher perceived stigma toward mental illness was also associated with WPV experience [57]. Within Cognitive Appraisal Theory, stigma functions as an environmental stressor shaping the meaning nurses attach to violent acts; where patients, families, and the wider community hold stigmatising views, nurses may appraise aggression as both less legitimate to report and less likely to elicit support, intensifying negative emotional responses and contributing to burnout and moral distress.

Consistent with the conceptual model, WPV exposure was associated with poorer quality of life across all WHOQOL-BREF domains and with lower job satisfaction (consistent with H2 and H3), indicating that the correlates of WPV extend beyond immediate safety incidents into broader occupational and functional well-being. The analytic strategy distinguished proximal occupational outcomes, including burnout, job satisfaction, and absenteeism, from distal outcomes, including quality of life and turnover intentions. Rather than implying causation, the observed pattern indicates that WPV exposure co-occurred with greater burnout and absenteeism, lower job satisfaction, poorer quality of life, and stronger intentions to leave. This pattern supports the relevance of examining WPV as a multidimensional occupational exposure with implications for both nurse well-being and workforce stability [58].

From a workforce perspective, the associations between WPV, absenteeism, and turnover intentions are notable (consistent with H1 and H3). WPV exposure and burnout were each associated with more days absent due to work-related stress or injury, indicating that violence is linked not only to subjective distress but also to behavioural withdrawal from work. Similarly, WPV exposure and burnout were positively associated with turnover intentions, whereas job satisfaction was negatively associated [59]. These associations are consistent with nurse-focused evidence showing that WPV and burnout are linked with turnover intentions across healthcare settings, and with Saudi workforce data identifying turnover and its antecedents as important concerns for the national nursing workforce [60].

Within the Transactional Model of Stress and Coping, these patterns suggest that when WPV is repeatedly appraised as threatening and insufficiently controlled, withdrawal, through sickness absence or consideration of leaving, may emerge as an understandable coping response. Because the design is cross-sectional, these findings describe associations rather than temporal pathways.

The mediation analysis (Hypothesis H4) provided additional insight into the statistical association between WPV exposure and turnover intentions. Burnout showed a partial statistical mediation pattern: WPV exposure was associated with higher burnout, and higher burnout was associated with stronger turnover intentions after accounting for WPV exposure. The indirect effect through burnout accounted for approximately 35.7% of the total association, while the direct association between WPV exposure and turnover intentions remained statistically significant.

This pattern is conceptually consistent with the Transactional Model of Stress and Coping and the Cognitive Appraisal Theory of Emotions. Recurrent WPV may be appraised as a persistent occupational stressor, co-occurring with exhaustion and reduced professional efficacy, which are in turn associated with stronger consideration of leaving the current position. However, because WPV, burnout, and turnover intentions were measured simultaneously, this finding should be interpreted as a statistical indirect association rather than evidence of a causal mechanism or temporal sequence. The persistence of the direct WPV–turnover association after accounting for burnout suggests that other factors, such as fear for personal safety, perceived organisational injustice, moral distress, or loss of trust in organisational protection, may also contribute to turnover intentions.

The cross-sectional design does not permit confirmation of temporal ordering, and reverse or bidirectional relationships cannot be excluded. Nonetheless, the observed mediation pattern provides a theoretically coherent and empirically supported hypothesis for future longitudinal and intervention research.

### 4.1. Implications for Occupational Health Practice, Psychiatric Nursing Management, and Policy

The findings carry implications for psychiatric nursing leadership, institutional policy, and national planning, framed as recommendations consistent with, rather than proven by, the present associations. First, WPV should be formally recognised as a correlate of psychiatric nurses’ quality of life and turnover intentions, and hospital and mental health authorities should mandate explicit prevention and response policies that include:

- Standardised, non-punitive incident-reporting systems with clear feedback and follow-up;
- Minimum staffing standards and systematic monitoring of nurse–patient ratios in high-acuity psychiatric units;
- Environmental and security measures, such as controlled access, alarm systems, and trained security personnel integrated into care teams.

Second, targeted support for high-risk groups is warranted. Early-career nurses and those repeatedly assigned to high-risk tasks should receive structured training in de-escalation, risk assessment, and self-protection, with an explicit focus on building de-escalation self-efficacy, and a systematic review of task-assignment practices (including the gendered distribution of high-risk duties) should ensure that exposure risk is not disproportionately concentrated. Third, organisations should implement structured post-incident pathways—mandatory debriefing after severe incidents, confidential psychological counselling, peer support, and supervision that addresses emotional and ethical responses, directly responsive to the observed association involving burnout. Fourth, because job satisfaction is an established correlate of nurse retention [42], investment in supportive, relationship-oriented nurse leadership may complement environmental and security measures. Finally, interventions to reduce stigma toward mental illness and toward help-seeking among healthcare workers are needed. Future research should prioritise longitudinal and mixed-methods designs to clarify temporal pathways, examine effects on patient outcomes and care quality, and test specific organisational and policy interventions.

### 4.2. Strengths, Limitations, and Future Directions

Several features strengthen confidence in the findings. The study achieved a high response rate and used a census approach that captured most psychiatric nurses providing direct care across three facilities. The major study constructs were measured using established instruments, with translation and cultural adaptation where necessary, and all multi-item scales demonstrated acceptable-to-excellent internal consistency in the present sample. The study also integrated a theory-informed conceptual model with bivariate analyses, multivariable regression, and statistical mediation, allowing WPV exposure to be examined in relation to both nurse well-being and workforce-related outcomes within a single analytic structure.

Several limitations should be considered when interpreting the findings. First, the cross-sectional design precludes conclusions about causality or temporal sequencing among WPV exposure, burnout, quality of life, absenteeism, job satisfaction, and turnover intentions; the mediation findings therefore represent statistical indirect associations rather than causal pathways. In addition, the a priori sample-size calculation was powered for the primary multivariable regression rather than for the indirect (mediation) effect; the precision of the mediation estimate is therefore limited, and the indirect effect should be regarded as preliminary. Second, all data were self-reported and collected in a single administration, raising the possibility of recall bias, social-desirability bias, and common method variance. Although Harman’s single-factor test did not indicate a dominant single-factor structure and confidentiality procedures may have reduced response bias, these risks cannot be fully excluded. Third, although the census approach achieved high participation, the sample size was moderate (N = 171) and drawn from one region of Saudi Arabia, limiting subgroup analyses and precluding formal interaction or moderation testing.

In particular, the relatively small number of nurses reporting no WPV exposure (n = 31) limits the precision of the logistic regression estimates for predictors of exposure, which should be interpreted with caution. Accordingly, contextual factors were examined as predictors, correlates, or covariates rather than as tested moderators. Fourth, generalisability to other Saudi regions, non-psychiatric settings, or healthcare systems with different staffing, security, and cultural conditions may be limited. Finally, the study-specific contextual-factors questionnaire demonstrated content validity and good internal consistency, but further psychometric evaluation, including confirmatory factor analysis, is warranted in larger and more diverse psychiatric nursing samples.

Future directions. Building on these findings, several priorities emerge for subsequent research. Longitudinal and cross-lagged designs are needed to establish the temporal ordering among WPV exposure, burnout, and turnover intentions and to test whether burnout operates as a genuine causal mechanism rather than a cross-sectional statistical mediator. Multi-region and multi-country studies would clarify the generalisability of the present associations across diverse staffing, security, and sociocultural contexts and would permit adequately powered tests of moderation (for example, by sex, career stage, or facility type) and of the indirect effect. Mixed-methods and qualitative work could illuminate the mechanisms underlying the elevated exposure observed among male and early-career nurses, including how high-acuity tasks are allocated in Saudi psychiatric settings. Finally, intervention and implementation research should evaluate whether multilevel prevention, safer staffing, and structured post-incident psychological support reduce burnout, protect quality of life, and improve retention, and should examine downstream effects on patient safety and care quality.

## 5. Conclusions

This study indicates that workplace violence is a pervasive occupational health and psychosocial safety concern for psychiatric nurses in Saudi Arabia. More than four in five nurses reported WPV in the preceding year, and WPV exposure was associated with higher burnout, lower job satisfaction, greater absenteeism, poorer quality of life, and stronger turnover intentions. These findings position WPV as an important correlate of both psychiatric nurses’ well-being and workforce stability. Situated within the Transactional Model of Stress and Coping and the Cognitive Appraisal Theory of Emotions, the findings are consistent with a pattern in which WPV exposure co-occurs with proximal occupational strain, poorer quality of life, and stronger intentions to leave. Burnout accounted for a substantial share of the statistical association between WPV exposure and turnover intentions, suggesting that burnout may be a potentially modifiable target for occupational health and retention strategies. Because the design is cross-sectional, the causal role of burnout in retention requires confirmation in longitudinal and intervention studies. With this caveat, multilevel violence prevention, safer staffing, and structured post-incident psychological support should be prioritised to protect psychiatric nurses’ well-being and support workforce retention.

## Figures and Tables

**Table 1:** Demographic and professional characteristics of psychiatric nurses (N = 171).

Variable	n (%)	Median (IQR)	Mean (SD)
Age (years)		35 (29–43)	36.2 (9.0)
20–30	45 (26.3)		
31–40	70 (40.9)		
41–50	40 (23.4)		
51–60	16 (9.4)		
Sex			
Male	30 (17.5)		
Female	141 (82.5)		
Marital status			
Single	60 (35.1)		
Married	100 (58.5)		
Divorced/Widowed	11 (6.4)		
Educational level			
Diploma	50 (29.2)		
Bachelor’s degree	100 (58.5)		
Master’s degree or higher	21 (12.3)		
Experience in psychiatric nursing (years)		8 (4–13)	8.5 (5.2)
<5 years	40 (23.4)		
5–10 years	70 (40.9)		
>10 years	61 (35.7)		
Facility type			
Comprehensive mental health complex	67 (39.2)		
Specialised psychiatric hospital	68 (39.8)		
Community-based mental health facility	36 (21.0)		

Note: Percentages are column percentages. Internal-consistency reliability for all multi-item scales is reported in Section 2.5.

**Table 2:** Prevalence and types of workplace violence experienced in the past 12 months (N = 171).

Type of WPV	n	%	95% CI
Verbal abuse	130	76.0	68.9–82.1
Threats	90	52.6	45.0–60.1
Intimidation	70	40.9	33.6–48.6
Physical assault	50	29.2	22.6–36.7
Any WPV (≥1 type)	140	81.9	75.4–87.0

Note: Categories are derived from the Workplace Violence Survey Questionnaire for the previous 12 months. Percentages are not mutually exclusive because nurses could report more than one type.

**Table 3:** Comparison of QoL, burnout, and job satisfaction by WPV exposure (N = 171).

Measure	WPV-Exposed (n = 140), Mean ± SD	Non-Exposed (n = 31), Mean ± SD	t	*p*	Cohen’s d
WHOQOL-BREF domains					
Physical health	60.5 ± 10.2	75.3 ± 9.8	−7.55	<0.001	1.46
Psychological	58.2 ± 11.5	73.8 ± 10.1	−7.58	<0.001	1.38
Social relationships	55.4 ± 12.3	70.1 ± 11.0	−6.58	<0.001	1.22
Environment	62.0 ± 9.5	77.4 ± 8.7	−8.77	<0.001	1.64
Overall QoL	59.0 ± 10.0	75.0 ± 9.5	−8.40	<0.001	1.61
Copenhagen Burnout Inventory					
Global burnout score (0–100)	47.9 ± 6.5	32.3 ± 6.0	12.93	<0.001	2.43
Job Satisfaction Survey (36–216)					
Total job satisfaction	130.5 ± 18.5	151.4 ± 17.0	−6.09	<0.001	1.15

Notes: Independent-samples *t*-tests compared nurses exposed to any WPV in the previous 12 months with those reporting no WPV exposure. Welch’s t-values are reported because of unequal group sizes. Cohen’s d was calculated using the pooled standard deviation; values are reported as absolute magnitudes (d ≥ 0.80 = large effect). Higher scores indicate better quality of life and job satisfaction; higher global burnout scores indicate greater burnout.

**Table 4:** Correlations between WPV exposure, quality of life, burnout, job satisfaction, absenteeism, and turnover intentions (N = 171).

Variable	WPV	QoL	Burnout	Job Sat.	Absent.	Turnover
WPV exposure	–	−0.65 **	0.72 **	−0.68 **	0.60 **	0.67 **
Quality of life	−0.65 **	–	−0.70 **	0.55 **	−0.58 **	−0.60 **
Burnout	0.72 **	−0.70 **	–	−0.62 **	0.65 **	0.70 **
Job satisfaction	−0.68 **	0.55 **	−0.62 **	–	−0.50 **	−0.58 **
Absenteeism	0.60 **	−0.58 **	0.65 **	−0.50 **	–	0.55 **
Turnover intentions	0.67 **	−0.60 **	0.70 **	−0.58 **	0.55 **	–

Note: ** *p* < 0.01 for all coefficients. Positive correlations indicate positive associations; negative correlations indicate inverse associations.

## Data Availability

The data supporting the findings of this study are not publicly available due to participant confidentiality and institutional data governance requirements, but are available from the corresponding author upon reasonable request.

## Supplementary Materials

The following supporting information can be downloaded at: https://www.mdpi.com/article/10.3390/healthcare14142190/s1, Table S1: Cross-tabulation of workplace violence exposure by demographic and professional characteristics among psychiatric nurses; Table S2: Logistic regression identifying demographic, professional, and contextual factors associated with any workplace violence exposure in the previous 12 months among psychiatric nurses; Table S3: Internal-consistency reliability of study instruments in the present sample; Table S4: Perceived contextual contributors to workplace violence among psychiatric nurses; Table S5: Covariate-adjusted multivariable regression models predicting absenteeism and turnover intentions.

## Abbreviations

The following abbreviations are used in this manuscript:

Abbreviation	Full term
CBI	Copenhagen Burnout Inventory
CI	Confidence Interval
IBM SPSS	IBM Statistical Package for the Social Sciences
ICN	International Council of Nurses
ILO	International Labour Organisation
IQR	Interquartile Range
JSS	Job Satisfaction Survey
PSI	Public Services International
QoL	Quality of Life
S-CVI	Scale-level Content Validity Index
SD	Standard Deviation
TIS-6	Turnover Intention Scale-6
WHO	World Health Organisation
WHOQOL-BREF	World Health Organisation Quality of Life—Brief Version
WPV	Workplace Violence
WVSQ	Workplace Violence Survey Questionnaire

## References

[1] O’Brien C.J., van Zundert A.A.J.J., Barach P.R. (2024). The Growing Burden of Workplace Violence against Healthcare Workers: Trends in Prevalence, Risk Factors, Consequences, and Prevention—A Narrative Review. EClinicalMedicine.

[2] Abdelmalik M., Alenezi A., Abdallah M., Ibrahim I., Ahmed M., Mohammead M.O., Mohammed A.M., Saeed A., Alhowaymel F.M., Abaoud A.F. (2025). Prevalence of Workplace Violence against Nurses in Saudi Arabia: A Systematic Review and Meta-Analysis. Int. J. Nurs. Sci..

[3] Abdulwehab S., Kedir F. (2025). Workplace Violence against Nurse: A Systematic Review and Meta-Analysis in Ethiopia. BMC Nurs..

[4] Chen M., Xie H., Liao X., Ni J. (2024). Workplace Violence and Turnover Intention among Chinese Nurses: The Mediating Role of Compassion Fatigue and the Moderating Role of Psychological Resilience. BMC Public Health.

[5] Tan L., Zheng S., Zhong X., Han Y., Xia L., Fan Y., He L. (2025). The Impact of Workplace Psychological Violence on Clinical Nurses’ Turnover Intention: The Mediating Role of Perceived Stress. Front. Public Health.

[6] Jallow A.D., Punpeng T., Boonshuyar C. (2024). The Prevalence and Risk Factors of Workplace Violence at a Psychiatric Hospital in The Gambia. Psychiatry Int..

[7] Eshah N., Al Jabri O.J., Aljboor M.A., Abdalrahim A., ALBashtawy M., Alkhawaldeh A., Saifan A., Ayed A., Rayan A. (2024). Workplace Violence Against Healthcare Workers: A Literature Review. SAGE Open Nurs..

[8] Gümüş C., Doğan M.B. (2025). The Impact of Workplace Violence on Healthcare Professionals: A Cross-Sectional Study for Understanding Professionalism and Burnout. Int. J. Ment. Health Nurs..

[9] Feruglio L., Bressan V., Cadorin L. (2025). Violence Against Nurses During Care: A Systematic Review. J. Clin. Nurs..

[10] Ibrahim I.A., Alenezi A., Abou Zeid M.A.G., Khedr M.A., El-Ashry A.M., Albagawi B.S., Abdelrahim S.M. (2025). Exploring the Role of Professional Burnout and Mental Health Strain in Nurses’ Turnover Intentions: A Mediation Study. J. Nurs. Manag..

[11] Beeber L.S., Delaney K.R., Martinez A.J.S. (2025). Keeping Our Patients and Ourselves Safe: Evidence-Based Knowledge to Prevent and Manage Violence in PMH Nursing Care. J. Am. Psychiatr. Nurses Assoc..

[12] Ramacciati N., Morales Palomares S. (2025). Violence towards Emergency Nurses: An Update of a Narrative Review of Theories and Frameworks. Int. Emerg. Nurs..

[13] Abu El-Kass S.M., Ellayan O.M., Turkman A.M., Al Mansour H.M., Alrowily M.A., Alsobhan K.A., Alruwaili B.A., Alqahtani N.S., Alruwaili H.A., El Bilbeisi A.H. (2025). The Prevalence of Workplace Violence toward Psychiatric Nurses in Saudi Arabia and Its Effect on Their Quality of Life. Front. Psychiatry.

[14] Elsharkawy N.B., Alruwaili A.N., Elsayed Ramadan O.M., Alruwaili M.M., Alhaiti A., Abdelaziz E.M. (2025). Barriers to Reporting Workplace Violence: A Qualitative Study of Nurses’ Perceptions in Tertiary Care Settings. BMC Nurs..

[15] Alhomoud F. (2025). “That’s Enough”-Workplace Violence Against Physicians, Pharmacists, and Nurses in Saudi Arabia: A Systematic Review of Prevalence, Causes, and Consequences. Risk Manag. Healthc. Policy.

[16] BinDhim N.F., Althumiri N.A., Al-Luhaidan S.M., Alhajji M., A Saad S.Y., Alyami H., Al-Duraihem R.A., Alhabeeb A.A. (2024). Assessing Attitudes toward Mental Health Illnesses in Saudi Arabia: A National Cross-Sectional Study. Int. J. Soc. Psychiatry.

[17] Alluhaibi B.A., Awadalla A.W. (2022). Attitudes and Stigma toward Seeking Psychological Help among Saudi Adults. BMC Psychol..

[18] El Khatib H., Alyafei A., Shaikh M. (2023). Understanding Experiences of Mental Health Help-Seeking in Arab Populations around the World: A Systematic Review and Narrative Synthesis. BMC Psychiatry.

[19] Wang H., Liu F., Ren Y., Sun Y. (2025). Workplace Violence, Career Identity, and Turnover Intentions: The Mediating Role of Job Burnout among Newly Recruited Rotational Nurses. BMC Nurs..

[20] Ammari N., Gantare A. (2025). Ethical Climate and Turnover Intention among Nurses: A Scoping Review. Nurs. Ethics.

[21] He J., Yang J., Yuan J., Yu Q., Stephano E.E., Zhang W., Li Y., Tian Y. (2026). Workplace Violence Against Nurses and Its Association With Mental Health and Turnover Intention: A National Cross-Sectional Study. J. Nurs. Manag..

[22] Al Mutair A., Al Bazroun M.I., Almusalami E.M., Aljarameez F., Alhasawi A.I., Alahmed F., Saha C., Alharbi H.F., Ahmed G.Y. (2022). Quality of Nursing Work Life among Nurses in Saudi Arabia: A Descriptive Cross-Sectional Study. Nurs. Rep..

[23] Xu D., Zhou J., Yang M., Yang L., Li Q., Zeng Y. (2025). Barriers and Facilitators to Reporting Workplace Violence among Healthcare Workers: A Mixed-Methods Systematic Review. Nurs. Outlook.

[24] Friese C.R., Medvec B.R., Marriott D.J., Khadr L., Wade M.G., Riba M., Titler M.G. (2024). Nurse-Reported Workplace Violent Events: Results from a Repeated Statewide Survey. Nurs. Outlook.

[25] Heijdra Suasnabar J.M., Finch A.P., Mulhern B., van den Akker-van Marle M.E. (2024). Exploring the Measurement of Health Related Quality of Life and Broader Instruments: A Dimensionality Analysis. Soc. Sci. Med..

[26] Kim S. (2023). World Health Organization Quality of Life (WHOQOL) Assessment. Encyclopedia of Quality of Life and Well-Being Research.

[27] Kellerer M., Süß S. (2026). Implications of Multiple Commitment Targets for Turnover Intentions and Actual Turnover: A Systematic Literature Review. Manag. Rev. Q..

[28] Alshmemri M. (2025). The Relationship Between Quality of Life and Nurses’ Turnover Intentions: A Cross-Sectional Study. J. Nurs. Manag..

[29] Ben-Zur H. (2019). Transactional Model of Stress and Coping. Encyclopedia of Personality and Individual Differences.

[30] Dewe P. (1997). The Transactional Model of Stress. Asia Pac. J. Hum. Resour..

[31] Lazarus R.S. (1991). Progress on a Cognitive-Motivational-Relational Theory of Emotion. Am. Psychol..

[32] Lazarus R.S., Folkman S. (1987). Transactional Theory and Research on Emotions and Coping. Eur. J. Pers..

[33] Khan U.S., Jain P.R. (2025). Research Designs and Methodologies. Introduction to Public Health and Research.

[34] Ahmed S.K. (2024). How to Choose a Sampling Technique and Determine Sample Size for Research: A Simplified Guide for Researchers. Oral Oncol. Rep..

[35] World Health Organisation (2003). Joint Programme on Workplace Violence in the Health Sector-Questionnaire. https://www.who.int/publications/m/item/workplace-violence-in-the-health-sector---country-case-study-research-instruments---survey-questionnaire.

[36] Skevington S.M.M., Lotfy M., O’Connell K.A.A. (2004). The World Health Organization’s WHOQOL-BREF Quality of Life Assessment: Psychometric Properties and Results of the International Field Trial. A Report from the WHOQOL Group. Qual. Life Res..

[37] World Health Organization (1998). WHOQOL-BREF User Manual. WHOQOL-BREF: Introduction, Administration, Scoring and Generic Version of the Assessment: Field Trial Version, December.

[38] Suárez L., Tay B., Abdullah F. (2018). Psychometric Properties of the World Health Organization WHOQOL-BREF Quality of Life Assessment in Singapore. Qual. Life Res..

[39] Kristensen T.S., Borritz M., Villadsen E., Christensen K.B. (2005). The Copenhagen Burnout Inventory: A New Tool for the Assessment of Burnout. Work Stress.

[40] Lani J., Spector P.E. (2010). Job Satisfaction Survey (JSS). https://www.statisticssolutions.com/free-resources/directory-of-survey-instruments/job-satisfaction-survey-jss/.

[41] Spector P.E. (1986). Assessing Employee Job Satisfaction with the Job Satisfaction Survey. https://paulspector.com/assessment-files/jss/jss-bibliography.doc.

[42] Luo Y., Zhang M., Yu S., Guan X., Zhong T., Wu Q., Li Y. (2024). The Impact of Psychological Violence in the Workplace on Turnover Intention of Clinical Nurses: The Mediating Role of Job Satisfaction. BMC Nurs..

[43] Bothma C.F.C., Roodt G. (2013). The Validation of the Turnover Intention Scale. SA J. Hum. Resour. Manag..

[44] Önal Ö., Evcil F.Y., Batmaz K., Çoban B., Doğan E. (2023). Systematic Review and Meta-Analysis of Verbal and Physical Violence against Healthcare Workers in the Eastern Mediterranean Region. East. Mediterr. Health J..

[45] Jang S.J., Son Y.J., Lee H. (2022). Prevalence, Associated Factors and Adverse Outcomes of Workplace Violence towards Nurses in Psychiatric Settings: A Systematic Review. Int. J. Ment. Health Nurs..

[46] Campbell T.S., Johnson J.A., Zernicke K.A. (2013). Cognitive Appraisal. Encyclopedia of Behavioral Medicine.

[47] Lim Z.Y., Idris D.R., Abdullah H.M.A.L., Omar H.R. (2023). Violence toward Staff in the Inpatient Psychiatric Setting: Nurses’ Perspectives: A Qualitative Study. Arch. Psychiatr. Nurs..

[48] Kafle S., Paudel S., Thapaliya A., Acharya R. (2022). Workplace Violence against Nurses: A Narrative Review. J. Clin. Transl. Res..

[49] Nam S., Wong J.Y.H., An B., Fong D.Y.T. (2025). Workplace Violence, Normalization of Violence, and Psychological Distress Among Korean Nurses: A Cross-Sectional Analytic Study. Int. Nurs. Rev..

[50] Spencer C., Sitarz J., Fouse J., DeSanto K. (2023). Nurses’ Rationale for Underreporting of Patient and Visitor Perpetrated Workplace Violence: A Systematic Review. BMC Nurs..

[51] Kim S., Mayer C., Jones C.B. (2021). Relationships between Nurses’ Experiences of Workplace Violence, Emotional Exhaustion and Patient Safety. J. Res. Nurs..

[52] Kolutek R., Erkutlu H., Chafra J. (2024). Workplace Violence and Nurses’ Psychological Well-Being: The Mediating Role of Burnout and the Moderating Role of Psychological Resilience. Arch. Psychiatr. Nurs..

[53] Çakır O., Akkoç İ., Arun K., Dığrak E., Uluırmak Ünlüeroğlugil H.S. (2025). The Complex Dynamics of Violence and Burnout in Healthcare: A Closer Look at Physicians and Nurses. Int. Nurs. Rev..

[54] Moran Jimenez J.D., Walden C., Carey A., Miller J., Keyes Young K., Morris L., Erbaio T. (2025). Seclusion Rates and Workplace Violence on a Psychiatric Emergency Department Unit. J. Am. Psychiatr. Nurses Assoc..

[55] Alarawi I.M.R., Alzeer F.G.H., Aljohani M.S.S., Aloofi M.M.B., Alsaedi S.E.N. (2025). Prevalence of Workplace Violence against Nurses in Psychiatric Hospital in Medina Region. Int. J. Innov. Res. Sci. Stud..

[56] Tong Y., Wang X., Lei C., Jian X., Xu X., Zheng Z., Ye Z., Ye Y. (2025). Workplace Bullying and Professional Identity among Male Nurses: The Mediating Role of Work Engagement and the Moderating Effect of Fear of Negative Evaluation. BMC Nurs..

[57] Al-Johani W.M., Al-Mansour A.H., AlBakr D.M., Alghirash D., Alfadhli A.N., Almutairi R., Mobarki O., Alqasim S., Al-Shammari M.A., Abdelwahab M.M. (2025). Stigma and Help-Seeking Attitudes in Relation to Psychological Distress Among Medical Students in Saudi Arabia. Clin. Pract. Epidemiol. Ment. Health.

[58] Yun S.H., Jun W.H. (2025). Structural Equation Model of Turnover Intention Among Integrated Nursing Care Service Wards Nurses in Small- and Medium-Sized Hospitals. West. J. Nurs. Res..

[59] Steffen P.R., Anderson T. (2025). Primary Appraisal Is Affective Not Cognitive: Exploring a Revised Transactional Model of Stress and Coping. Appl. Psychophysiol. Biofeedback.

[60] Yang H.L., Tai J.C., Wang C.H., Shyu Y.K., Chou K.R., Pien L.C. (2025). Workplace Violence, Work Characteristics, and Seniority Levels among Nurses: A Cross-Sectional Study. BMC Nurs..

