# Supplementary material for: Workplace Violence as an Occupational Hazard in Psychiatric Nursing: Burnout, Quality of Life, and Turnover Intentions in Saudi Arabia
Source: Healthcare (Basel). 2026 Jul 20;14(14):2190. doi: 10.3390/healthcare14142190 (PMC13411834; doi:10.3390/healthcare14142190)
Supplement: Supplementary file 1 [file healthcare-14-02190-s001.zip › healthcare-4344529-supplementary.pdf]

Supplementary Tables

Table S1. Cross-tabulation of workplace violence exposure by demographic and professional characteristics among psychiatric nurses (N = 171).

Variable	Category	Any WPV n (%)	Physical assault n (%)	Verbal abuse n (%)	Threats n (%)	Intimidation n (%)
Age group	20–30 years (n = 45)	41 (91.1)	18 (40.0)	41 (91.1)	30 (66.7)	26 (57.8)
	31–40 years (n = 70)	58 (82.9)	20 (28.6)	54 (77.1)	35 (50.0)	28 (40.0)
	41–50 years (n = 40)	31 (77.5)	9 (22.5)	26 (65.0)	18 (45.0)	12 (30.0)
	51–60 years (n = 16)	10 (62.5)	3 (18.8)	9 (56.3)	7 (43.8)	4 (25.0)
Sex	Male (n = 30)	28 (93.3)	15 (50.0)	27 (90.0)	22 (73.3)	19 (63.3)
	Female (n = 141)	112 (79.4)	35 (24.8)	103 (73.0)	68 (48.2)	51 (36.2)
Psychiatric nursing experience	<5 years (n = 40)	37 (92.5)	16 (40.0)	36 (90.0)	28 (70.0)	24 (60.0)
	5–10 years (n = 70)	56 (80.0)	18 (25.7)	54 (77.1)	36 (51.4)	28 (40.0)
	>10 years (n = 61)	47 (77.0)	16 (26.2)	40 (65.6)	26 (42.6)	18 (29.5)

Note: Cell values represent the number and percentage of nurses in each subgroup reporting at least one incident of the specified WPV type during the previous 12 months. Percentages are calculated within each subgroup. Categories of WPV are not mutually exclusive because participants could report more than one type of violence. WPV = workplace violence.

This descriptive table shows that any WPV exposure was proportionally higher among younger nurses, male nurses, and nurses with fewer than five years of psychiatric nursing experience. This pattern is consistent with the multivariable logistic regression findings reported in Supplementary Table S2.

Table S2. Logistic regression identifying demographic, professional, and contextual factors associated with any workplace violence exposure in the previous 12 months among psychiatric nurses (N = 171).

Predictor	Odds ratio (OR)	95% CI	p
Demographic and professional factors			
Age 20–30 years vs. 51–60 years	2.50	1.20–5.20	0.015
Male sex vs. female sex	2.00	1.10–3.65	0.025
<5 years vs. >10 years of psychiatric nursing experience	3.00	1.40–6.45	0.005
Systemic/organisational factors			
Lower perceived staffing adequacy vs. higher perceived staffing adequacy*	2.00	1.18–3.33	0.012
Inadequate vs. adequate security measures*	2.22	1.25–4.00	0.006
Sociocultural factor			
Higher vs. lower perceived stigma toward mental illness*	1.70	1.10–2.60	0.015

Notes: *Contextual factors were dichotomised at the sample median. OR > 1 indicates higher odds of reporting any WPV exposure. The model included age, sex, psychiatric nursing experience, facility, staffing adequacy, security measures, and perceived stigma toward mental illness. CI = confidence interval; OR = odds ratio; WPV = workplace violence.

Younger age, male sex, shorter psychiatric nursing experience, lower perceived staffing adequacy, inadequate security, and higher perceived stigma toward mental illness were independently associated with higher odds of reporting any WPV exposure.

Table S3. Internal-consistency reliability of study instruments in the present sample (N = 171).

Instrument/subscale	No. of items	Cronbach's α
Workplace Violence Survey Questionnaire (WVSQ) — total	32	0.93
Physical assault	8	0.90
Verbal abuse	8	0.88
Threats	8	0.85
Intimidation	8	0.84
WHOQOL-BREF — overall	26	0.89
Physical health	7	0.82
Psychological	6	0.84
Social relationships	3	0.74
Environment	8	0.86
Copenhagen Burnout Inventory (CBI) — global score	19	0.94
Personal burnout	6	0.90
Work-related burnout	7	0.87
Client-related burnout	6	0.83
Job Satisfaction Survey (JSS) — total	36	0.91
Turnover Intention Scale-6 (TIS-6)	6	0.87
Contextual-factors questionnaire — total	45	0.90
Cultural factors	15	0.82
Social factors	15	0.81
Systemic/organisational factors	15	0.88

Note: Cronbach's α values ≥0.70 were interpreted as indicating acceptable internal consistency. The CBI global score was calculated on the conventional 0–100 metric as the mean of the three domain scores. CBI = Copenhagen Burnout Inventory; JSS = Job Satisfaction Survey; TIS-6 = Turnover Intention Scale-6; WVSQ = Workplace Violence Survey Questionnaire.

All study instruments demonstrated acceptable-to-excellent internal consistency in the present sample, supporting the internal consistency of the scale scores used in subsequent analyses.

Table S4. Perceived contextual contributors to workplace violence among psychiatric nurses (N = 171).

Contributing factor	Mean ± SD (1–5)
Cultural factors	
Stigma toward mental illness	3.8 ± 0.9
Societal attitudes	3.5 ± 1.0
Family involvement during psychiatric crises	3.2 ± 1.1
Social factors	
Communication barriers	3.6 ± 0.8
Patient demographics and acuity	3.4 ± 0.9
Limited community support	3.1 ± 1.0
Systemic/organisational factors	
Staffing insufficiency	4.0 ± 0.7
Security limitations	3.7 ± 0.8
Workplace policy limitations	3.5 ± 0.9
Limited training on WPV prevention	3.3 ± 1.0
Limited institutional support	3.2 ± 1.1

Note: Scores are presented as mean ± standard deviation on a 5-point Likert scale. Higher scores indicate stronger perceived contribution to WPV risk. SD = standard deviation; WPV = workplace violence. Representative highest-rated items within each domain are shown; the full contextual-factors questionnaire comprised 45 items (15 per domain, rated 1–5).

Staffing insufficiency, security limitations, and stigma toward mental illness had the highest mean ratings, indicating that participants perceived organisational protection and sociocultural context as important contributors to WPV risk.

Table S5. Covariate-adjusted multivariable regression models predicting absenteeism and turnover intentions (N = 171).

Model / Predictor	B	95% CI	p
Model A: Absenteeism, adjusted linear regression model			
WPV exposure	0.33	0.13–0.53	<0.001
Global burnout score	0.69	0.21–1.17	0.004
Model B: Turnover intentions, adjusted linear regression model			
WPV exposure	0.38	0.20–0.56	<0.001
Global burnout score	0.84	0.42–1.26	<0.001
Job satisfaction	−0.24	−0.40 to −0.08	0.003

Models were re-estimated with adjustment for age, sex, years of psychiatric nursing experience, and facility. Model A is presented as a covariate-adjusted linear sensitivity model for absenteeism; the primary count-model approach is described in the main manuscript. Global burnout was analysed on the conventional CBI 0–100 metric. B = unstandardised coefficient; CI = confidence interval; WPV = workplace violence.

After adjustment for age, sex, years of psychiatric nursing experience, and facility, WPV exposure and global burnout remained statistically associated with absenteeism and turnover intentions. Job satisfaction also remained inversely associated with turnover intentions, supporting the robustness of the parsimonious models reported in the main manuscript.
